# Cancer Vaccines, Adjuvants, and Delivery Systems

**DOI:** 10.3389/fimmu.2021.627932

**Published:** 2021-03-30

**Authors:** Samantha J. Paston, Victoria A. Brentville, Peter Symonds, Lindy G. Durrant

**Affiliations:** ^1^ Biodiscovery Institute, Scancell Limited, Nottingham, United Kingdom; ^2^ Biodiscovery Institute, University of Nottingham, Faculty of Medicine and Health Sciences, Nottingham, United Kingdom

**Keywords:** adjuvant, peptide vaccine, DNA vaccine, cancer, vaccine

## Abstract

Vaccination was first pioneered in the 18th century by Edward Jenner and eventually led to the development of the smallpox vaccine and subsequently the eradication of smallpox. The impact of vaccination to prevent infectious diseases has been outstanding with many infections being prevented and a significant decrease in mortality worldwide. Cancer vaccines aim to clear active disease instead of aiming to prevent disease, the only exception being the recently approved vaccine that prevents cancers caused by the Human Papillomavirus. The development of therapeutic cancer vaccines has been disappointing with many early cancer vaccines that showed promise in preclinical models often failing to translate into efficacy in the clinic. In this review we provide an overview of the current vaccine platforms, adjuvants and delivery systems that are currently being investigated or have been approved. With the advent of immune checkpoint inhibitors, we also review the potential of these to be used with cancer vaccines to improve efficacy and help to overcome the immune suppressive tumor microenvironment.

## Introduction

The potential to develop a cancer vaccine has been extensively researched in both humans and animal models, the majority of these vaccines are therapeutic vaccines that aim to activate the immune system to recognize and kill established tumors. A prophylactic vaccine that prevents cancers caused by the Human Papillomavirus [Types 6, 11, 16, 18] has been approved and in the UK, children aged 12-13-years-old are routinely offered this vaccine. Developing a therapeutic cancer vaccine has been more problematic with many encouraging results in preclinical studies not translating into the clinic. To date the FDA has only approved one vaccine, sipuleucel-T, that is used to treat metastatic castration-resistant prostate cancer in a limited group of nearly asymptomatic patients (1). There are number of reasons for these failures such as the immune suppressive TME, lack of a robust T cell responses, sub optimal vaccine formulations, delivery, adjuvants and identifying the best patients to target. The ideal setting for a cancer vaccine to work is in patients following surgical resection, chemotherapy or radiotherapy, all of which stimulate an immune response themselves. Vaccination at this stage and in combination with a checkpoint inhibitor will provide the best setting to induce a potent anti-tumor immune response.

Over the last couple of decades, a better understanding of the tumor microenvironment (TME) and immune suppressive mechanisms have opened a number of new avenues that can be explored and has led to the next generation of new cancer therapies. The immune suppressive TME is a major obstacle to the success of any cancer vaccine with the description of immunologically “cold” tumors that on their own do not appear to be immunogenic with an absence of tumor infiltrating lymphocytes (TILs). In contrast “hot” tumors are immunogenic and have induced an immune response that has resulted in the infiltration of TILs but are not able to function due to the presence of various checkpoint molecules such as PD-1, CTLA-4, LAG-3, TIM-3 or the presence of immune suppressive cells such as regulatory T cells, myeloid-derived suppressor cells (MDSCs), M2 macrophages, regulatory natural killer (NK) T cells or cytokines such as transforming growth factor-beta [TGFβ], IL-10, and IL-13 (2, 3). The success of any cancer vaccine relies on overcoming the immune suppressive TME and converting “cold” tumors into “hot” tumors and therefore inducing a robust tumor specific immune response that can kill cancer cells.

## Target Antigens

The choice of antigen to target in any cancer vaccine is extremely important to the efficacy of the vaccine in the clinic. The ideal antigen should be specifically expressed on cancer cells with no expression on normal cells, ideally the antigen should be necessary for cell survival and be highly immunogenic.

Tumor antigens fall into two broad categories, the tumor associated antigens (TAAs) and tumor-specific antigens (TSAs). Within each category a number of different types of tumor antigens have been described and are summarized in **Table 1**. The cancer germline antigens (also called cancer testis antigens) are the most studied group of cancer antigens, historically they were attractive antigens to target due to their expression only on germ cells of immune-privileged organs and high expression on tumor cells. The most common cancer germline antigens that are targeted include MAGE (4, 5), NY-ESO1 (6), GAGE (7, 8) and BAGE (9), both T cell and antibody responses to these antigen have been detected in patients.

**Table 1:** Different types of tumor antigens.

	Class of tumor antigen	Description	Tumor specificity	Example of tumor antigen
**Tumor Specific Antigens (TSA)**	Cancer Germline antigens	Expression on healthy cells limited to testes, fetal ovaries and trophoblasts. Expressed on a wide a variety of cancer types.	High	MAGE, NY-ESO-1, GAGE, BAGE
	Tumor specific mutated antigens	Mutations resulting in the generation of a new peptide. Mutations can be generated at the gene level, from chromosome translocations or due post translational modification.	High	KRAS, p53, NRAS, BCR-ABL translocation, ETV6, NPM/ALK, ALK.
	Oncogenic viral antigens	Abnormal expression on cells infected with an oncovirus.	High	EBV LMP-1/LMP-2A, HPV E6/E7, HTLV-1 Tax
**Tumor associated Antigens (TAA)**	Tissue Differentiation antigens	Antigen expressed on tumor cells and normal cells.	Low	Melan A/MART-1, gp100, Tyrosinase, PSA, CEA.
	Overexpressed antigens	Antigen over expressed on tumor cells and normal level of expression on healthy cells.	Low	HER2, hTERT, p53, Survivin, MUC1, WT1, cyclin B.

The differentiation antigens are expressed on normal and tumor cells from the same tissue, targeting such antigens requires careful consideration to any potential toxicity to the normal tissue. Differentiation antigens include the melanoma antigens Melan A/MART-1 (10, 11), gp100 (12), tyrosinase (13), the prostate antigen prostate specific antigen (PSA) (14, 15) and the colon antigen carcinoembryonic antigen (CEA) (16, 17).

The overexpressed antigens are generally expressed at low levels on normal cells but are over expressed on tumor cells, there are many antigens that fall into this group including HER2 (18), hTERT (19, 20), p53 (21), survivin (22–25), MUC1 (26), WT1 (27), cyclin B (28, 29) and many more. Targeting over expressed antigens can be challenging, preclinical studies need to ensure that normal low-level expressing cells are not targeted by the vaccine induced immune response.

The oncogenic viral antigens are expressed on virus infected cells that have subsequently undergone malignant transformation. Oncogenic viral antigens have been targeted in both prophylactic vaccines such as HPV but also in therapeutic vaccines to treat existing malignancies. The most commonly targeted oncogenic viral antigens in this group include EBV LMP-1 and LMP-2A (30–32), HPV E6/E7 (33), HTLV-1 Tax (34).

The last group of cancer antigens are those antigens that are mutated, these mutations can be generated at the gene level or as a result of post translational modifications leading to the generation of a new peptide. In the last couple of years there has been a renewed effort in generating vaccines that target mutated antigens, in particular the neoantigens. There are very few mutated antigens described where the mutated peptide is shared across patients or cancer types, the most studied shared mutations are KRAS* * (35), NRAS (36), epitopes from BCR-ABL translocation (Chronic myeloid leukemia) (37, 38), ETV6 (acute myeloid leukemia) (39), NPM/ALK (anaplastic large cell lymphomas) (40, 41) and ALK (neuroblastoma) (42, 43). A number of groups are developing personalized vaccines that target neoantigens identified from the patient’s tumor, very few if any of these mutations are shared epitopes and therefore any generated vaccine is only specific to the individual. Neoantigens are immunogenic because they harbor mutations, they have escaped central tolerance and are recognized as “non self” by the adaptive immune system (44). Despite the higher immunogenicity of neoantigen’s only 1-2% of T cells recognize these antigens (45). The poor immunogenicity of many tumors means that designing an effective neoantigen tumor vaccine will need to overcome these challenges.

The post translational modified cancer antigens are another group of antigens, they are not subject to thymic deletion and are therefore attractive vaccine candidates. A number of different post-translational modifications have been described that generate tumor specific epitopes including glycopeptides (46), phosphopeptides (47, 48) and citrullinated peptides (49). Cancer cells often exhibit different phosphorylation patterns leading to the generation of phosphorylated antigens, these make attractive vaccine candidates (47, 48, 50). Phosphorylated epitopes can be naturally processed and presented on the cell surface in association with MHC class I molecules for recognition by CD8+ T cells (50–52). Unregulated signaling cascades in tumor cells often lead to an increase in protein phosphorylation within the cell which in turn leads to the generation of phosphopeptides (52). Phosphopeptides have been identified by mass spectrometry analysis of tumor biopsies and cancer cell lines (53). Engelhard et al. (53) identified two phosphorylated peptides derived from the insulin receptor substrate 2 (IRS2) protein and breast cancer anti-estrogen resistance 3 (BCAR3). The ISR2 protein is overexpressed in many cancer types and *in vivo* has been shown to enhance metastasis (54–56), BCAR3 is associated cellular migration and resistance to therapeutic anti-estrogens in breast cancer cells (57, 58). Phosphopeptides restricted by HLA-*02:01 were identified by mass spectrometry and included in a phase 1 clinical trial (NCT01846143) in patients with resected stage IIA–IV melanoma. All patients had treatment related adverse events, but none were grade 3-4, T cell responses were induced to the phosphorylated IRS2 (1097-1105) peptide in 5/12 patients and to the phosphorylated BCAR3 (126-134) peptide in 2/12 patients. This trial showed that phosphopeptides are safe and induced an immune response in some patients, however, with the advent of immune checkpoint inhibitors future studies will need to define and enhance the immune response induced to these peptides.

**Figure 1:**
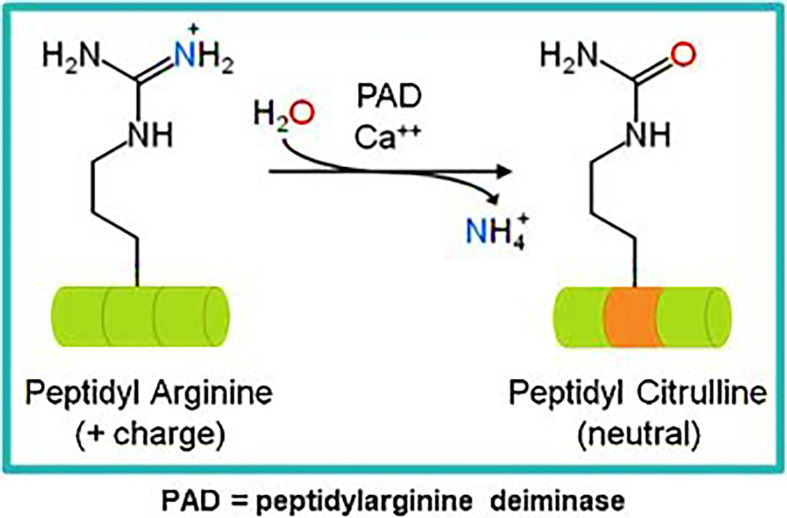
Schematic of the citrullination or deamidation of arginine.

**Figure 2:**
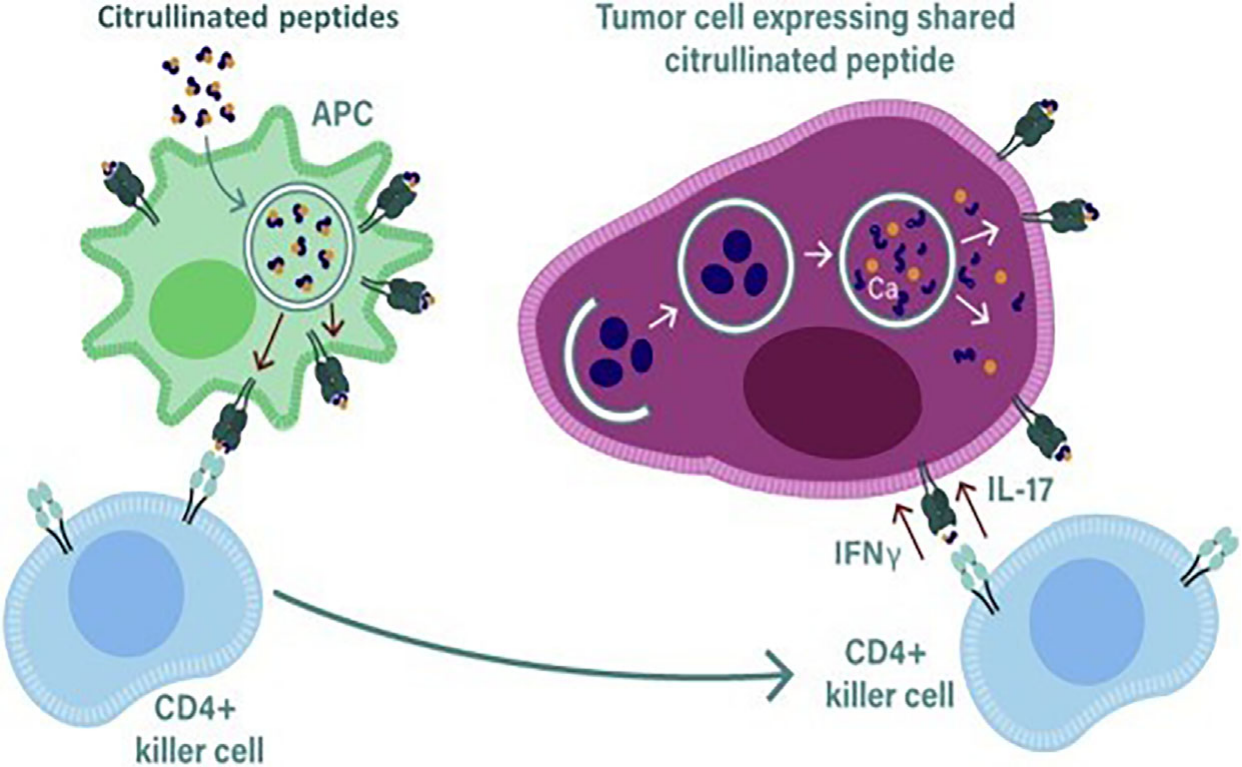
During stress induced autophagy and in the presence of inflammation citrullinated peptides can be presented on major histocompatibility complex (MHC) class II molecules for recognition by CD4+ T cells. During inflammation many cytokines are produced, the majority are proinflammatory that result in the upregulation of MHC class II expression that then activates CD4+ T cells. Primed killer CD4 T cells enter the tumor and are reactivated by APCs presenting citrullinated peptides from tumors allowing recognition and lysis by the killer CD4 T cells.

Our own research has focused on epitopes that are citrullinated in tumor cells. Citrullination is a post translation modification where positive charged arginine residues are converted into neutrally charged citrulline in a process catalyzed by the Ca^2+^ dependent peptidyl arginine deaminase (PADI) enzymes (59, 60) (**Figure 1**). This modification can impact the protein structure and induce changes that result in protein denaturation potentially altering the structure and the function of the protein (61, 62). We have detected T cell responses to citrullinated peptides in healthy donors (60) suggesting that the T cells recognizing them are positively and not negatively selected in the thymus. In healthy cells the PADI enzymes are maintained in an inactive state due to low concentrations of Ca^2+^ (34), in double membrane vesicles within viable cells the calcium concentrations can be high leading to the activation of the PADI enzymes. Citrullination can occur within autophagosomes as a result of autophagy, here high calcium levels activate PADI enzymes that then citrullinate engulfed proteins from the cytoplasm (36, 37), this process is induced in stressed cells (17) such as cancer cells. During stress induced autophagy and in the presence of inflammation citrullinated peptides can be presented on major histocompatibility complex (MHC) class II molecules for recognition by CD4+ T cells (63). During inflammation many cytokines are produced, the majority are proinflammatory that result in the upregulation of MHC class II expression that then activates CD4+ T cells (**Figure 2**).

A number of studies performed in autoimmune patients have demonstrated that CD4+ T cell responses can be detected to citrullinated proteins such as the intermediate filament protein vimentin and the glycolytic enzyme enolase (64–68). In ovarian cancer patients we have demonstrated the presence of CD4+ T cell responses to citrullinated peptides derived from α-enolase and vimentin (60). The constitutive expression of MHC class II is mainly restricted to APCs such as DCs, B cells and macrophages but other cells such thymic epithelia cells and activated T cells can also express MHC class II (69). The expression of MHC class II on most other cells can be induced by interferon gamma (IFNγ) present in the local vicinity. The expression of MHC class II is controlled by the Class II Major Histocompatibility complex transactivator (CIITA) which is regulated by four different promoters, promoter I is active in myeloid cells, promoter III in lymphocytes and promoter IV is necessary for responsiveness to IFNγ (70), the function of promoter II is unknown, transcripts from this promoter are rare and therefore its function has not been pursued. Most tumors in the presence of inflammation and IFNγ will express MHC class II, if citrullinated peptides are then generated in response to stress or autophagy these can then be loaded onto MHC class II for presentation on the surface of tumor cells.

We have focused on citrullinated vimentin and α-enolase as attractive cancer vaccine targets. Vimentin is an intermediate filament protein that is known to be citrullinated and overexpressed in a wide range of cancers (71–76), particularly during EMT (77). The glycolytic enzyme α-Enolase (ENO1) catalyzes the final step in glycolysis (78). Many tumors switch to generating their energy *via* glycolysis in a process termed the “Warburg effect” and therefore overexpress ENO1, a wide range of tumors overexpress ENO1 (79–82). Due to its ubiquitous expression, ENO1 is often degraded during autophagy; previous studies have also shown that ENO1 can be citrullinated (65, 83). We have shown that these citrullinated peptides are recognized and presented to CD4+ T cells by both MHC class II HLA-DR4 and HLA-DP4 molecules (49, 84, 85). HLA transgenic mice vaccinated with citrullinated vimentin and α-enolase peptides linked to an adjuvant (Modi-1 vaccine) can stimulate CD4+ T cells (49, 64, 86) and generate potent anti-tumor responses resulting in tumor regression and eradication with no associated toxicity (49, 87). We have also shown that healthy donors have a repertoire of T cells that can be detected following stimulation with the citrullinated vimentin and α-enolase peptides showing that citrullinated peptides can be presented in the thymus allowing positive selection and resulting in specific T cell repertoires capable of recognizing these peptides (87). Our preclinical data shows that citrullinated vimentin and α-enolase are promising candidate vaccine targets and as a vaccine have generated impressive anti-tumor responses in preclinical murine models.

## Adjuvants

Antigens alone in a vaccine are poor inducers of the adaptive immune response. In the absence of an adjuvant antigens targeted to immature DCs in the absence of inflammation or any microbial stimulation induce tolerance instead of a potent immune response (88). Adjuvants need to attract immune cells to the site of injection while also promoting cell mediated trafficking of antigen to draining lymph nodes and triggering the activation of APCs.

### Current Vaccine Adjuvants

The water-in-oil emulsions such as Montanide ISA 720 and Montanide ISA-51 have been widely adopted as adjuvants, they form a depot at the injection site, this results in the trapping of the soluble antigens preventing their rapid trafficking to local lymph nodes, this induces inflammation and the gradual release of the antigen. In a clinical trial Montanide ISA-51 was shown to induce both CD4+ and CD8+ T cell responses in patients vaccinated with long peptides of the oncoproteins E6 and E7 (89).

New vaccine adjuvants have been developed that target specific components of the immune system to generate a more robust and longer lasting immune response. Newer adjuvants that consist of Pathogen-associated molecular pattern molecules (PAMPs) are now being used, these provide a danger signal that is recognized by pattern recognition receptors (PRRs) inducing an immune response. Innate cells express PPRs, these receptors include the Toll-like receptors (TLRs), nucleotide binding oligomerization domain like receptors and the mannose receptor. TLR agonists are increasingly being used as a vaccine adjuvant, they mimic microbial stimulation and have been shown to increase vaccine efficacy (90) particularly for cancers (91). Lymph node targeted TLR agonists have shown a direct relationship between the magnitude of CD8+ T cell responses and the amount of TLR agonist accumulated in draining lymph nodes, demonstrating the importance of providing sufficient inflammatory signals during immunization (92). A number of TLR agonists are currently in trial as adjuvants for cancer vaccines, one of the most commonly used TLR agonist is polyinosinic–polycytidylic acid with polylysine and carboxymethylcellulose (Poly-ICLC) a TLR3 agonist (93), others include monophosphoryl lipid A (MPLA) a TLR4 agonist (94, 95), imiquimod a TLR7 agonist (96, 97), resiquimod a TLR7 and TLR8 agonist (98, 99), CpG oligodeoxynucleotide (CpG ODN) a TLR9 agonist (90, 100, 101).

### New Emerging Vaccine Adjuvants

Other newer adjuvants are also being investigated to increase the efficacy of a cancer vaccine, these include the CD40 agonists, these directly target the antigen to the early endosomes of DCs and mediate cross presentation. Although CD40 agonist antibodies have not been extensively studied in clinical trials as a vaccine adjuvant, they have been studied independently as monotherapy (102). A number of preclinical mouse models have shown that CD40 agonists can be used in combination with TLR agonists in a vaccination strategy (103, 104), whether this translates into clinical efficacy is still to be determined.

Another class of potential adjuvants is the Stimulator of interferon genes protein (STING) agonists. STING is a transmembrane protein located in the endoplasmic reticulum (105), its activation triggers a type I interferon response in response to intracellular DNA (106). STING agonists include synthetic cyclic dinucleotide derivatives and cyclic di-guanosine monophosphate, these have all shown anti-tumor activity in mice (107, 108). STING expression is highest on T cells and STING activation can lead to T cell apoptosis, such effects are not seen with macrophages and DCs (109). To use a STING agonist in a cancer vaccine it would need to be combined with an adjuvant or delivery system that targets only myeloid cells *in vivo* (110) preventing T cell apoptosis. STING agonists do induce some systemic toxicity and to overcome this intratumoral injection is the preferred route of administration. In addition, preclinical studies of STING agonists have been complicated by their differential binding properties in murine and human cells (111). The potential toxicity of STING agonists and lack of specific targeting could limit their use as adjuvants in a cancer vaccine.

In addition to using pathogen derived molecules as adjuvants a number of cytokines have also been shown to act as an adjuvant. Immunostimulatory cytokines such as IL-2 (112, 113), IFN (114), IL-12 (115, 116) and granulocyte-macrophage colony stimulating factor (GM-CSF) (117–119) have all been investigated, although recent studies have focused mainly on their application in cellular based therapies and vaccines. GM-CSF is the most studied immunostimulatory factor and has been included in numerous cancer vaccine trials (120). In preclinical studies GM-CSF looked a very promising candidate, it helps recruit DCs to the injection site, it can promote the maturation of DCs and antigen presentation resulting in an enhanced adaptive immune response (117). However, in clinical trials GM-CSF has generated disappointing results with only a few trials having shown a clinical benefit, the results across the majority of trials have been inconsistent. Preclinical studies indicated that GM-CSF could expand MDSCs resulting in the suppression of cell mediated anti-tumor responses (118). The effect of GM-CSF was also observed in clinical trials where a low dose of GM-CSF induced the expansion of CD14 positive, HLA-DR low/negative myeloid cells. In another study GM-CSF was used with incomplete Freund’s adjuvant and resulted in a low T cell response when compared to vaccine adjuvant without GM-CSF (119). Despite these results a number of clinical trials are currently underway using GM-CSF as an adjuvant component.

We have previously described our Modi-1 peptide vaccine (60), this vaccine comprises of two citrullinated vimentin peptides, as well as a citrullinated peptide from α-enolase, each peptide is conjugated to the TLR1/2 ligand adjuvant AMPLIVANT^®^ (ISA Pharmaceuticals BV, Leiden, the Netherlands). In preclinical studies we have shown that by combining a peptide vaccine with a TLR ligand adjuvant promotes a Th1 response that is capable of inducing a potent anti-tumor response in tumor bearing mice (60). The CD4+ but not CD8+ T cells were essential for the generation of the anti-tumor response, depleting CD4+ T cells abrogated this response and a corresponding increase in CD4+ tumor-infiltrating lymphocytes (TILs) was associated with tumor regression (60). A comparison of different Toll-like receptor (TLR)-stimulating adjuvants showed that Modi-1 induced strong Th1 responses when combined with GM-CSF, TLR9/TLR4, TLR9, TLR3, TLR1/2 and TLR7 agonists. The strongest response was observed with TLR1/2 AMPLIVANT^®^ adjuvant. The AMPLIVANT^®^ adjuvant is already being used in an ongoing study evaluating two HPV-16 peptides in patients with head and neck squamous cell carcinoma (NCT02821494). These results highlight the importance of screening a range of adjuvants and doses to find the optimal adjuvant and dose to induce a potent immune response. The Modi-1 vaccine will enter a Phase 1/2 clinical trial in 2021.

## Delivery Systems

### Electroporation and Gene Gun Vaccine Delivery

There have been significant improvements in optimizing vaccine administration routes to overcome poor cellular uptake. Also improvements with delivery and plasmid design have improved the efficacy of DNA vaccines in both pre-clinical and clinical studies (121). One strategy for improving the uptake of plasmid DNA into antigen presenting cells (APCs) is by using electroporation. Electroporation delivers small electrical pulses that causes transient pores to form in the cell membrane. During the period of membrane destabilization plasmid DNA present in the extracellular environment around the target cell gains access to the intracellular compartment (122). Following the transfer of DNA into the cell the membrane then reseals. The transient increase in the permeability of the target cell membrane enhances the uptake of plasmid DNA (123). Electroporation increases DNA uptake by over a 1000-fold and has an adjuvant effect due to local tissue damage and the resulting stimulation of proinflammatory cytokine in the local vicinity (124, 125). A number of DNA vaccines currently in clinical trials are using electroporation to delivery DNA plasmids, the ability of these plasmids to induce an immune response has been demonstrated in prostate cancer and melanoma (126). Another similar strategy is using a gene gun to deliver plasmid DNA that is coated with a heavy metal, typically gold particle are used, APCs at the injection site are bombarded with plasmid coated particles. The gene gun strategy reduces the amount of DNA required by 100-1,000 (127); some promising preclinical data has led to phase 1 and 2 clinical trials in head and neck squamous cell carcinoma and cervical cancer (128). Electroporation is also being used to deliver plasmid DNA in the infectious disease field, there are a number of COVID-19 DNA vaccines currently in in clinical trial (WHO landscape report Dec 2020) that use DNA plasmids encoding the Spike antigen and using electroporation as a delivery system (NCT04445389, NCT04447781, NCT04642638, NCT04627675). The main disadvantages of a DNA vaccine is when electroporation is used as a delivery system, electroporation can cause considerable pain and anxiety on administration and not suitable for mass vaccination programs, alternative delivery systems are currently being pursued.

### Nanoparticle Vaccine Delivery Systems

Nanoparticle based drug delivery platforms offer an alternative vehicle for delivering drugs that have previously suffered from pharmacokinetic limitations including poor bioavailability, a short half-life or poor solubility. A variety of nanoparticles have been explored as delivery systems or as adjuvants, such as polymeric nanoparticles, liposomes, micelles, carbon nanotubes, mesoporous silica nanoparticles, gold nanoparticles and virus nanoparticles, that can all be used alone or in combination (129). Liposomes are a popular nanoparticle vaccine delivery system, they are versatile and can be constructed with a variety of different properties by changing the lipid composition, charge, size and surface properties (130–132). Nanoparticle based drug delivery platforms use well-known lipid carriers to deliver biotherapeutic encoding tumor antigens directly into APCs such as dendritic cells. The targeting of APCs in the lymphoid compartments is accomplished by using well-known lipid carriers such as DOTMA, DOTAP, DOPE and cholesterol and by adjusting negative net charge of the nanoparticles to provide optimal drug delivery. Cationic liposomes are mainly composed of the lipids DOTMA and DOPE that form colloidally stable nanoparticles of reproducible particle size (200–400 nm) with an excess of positive charge preventing excessive aggregation (133). Liposomes can increase the immunogenicity of target antigens for cancer vaccines and have been used to deliver RNA, DNA and antigens. Hydrophilic and lipophilic antigens can be loaded into liposomes, the hydrophilic antigens are trapped in the aqueous inner space and the lipophilic components are inserted into the lipid bilayer by adsorption or chemical attachment. Liposomes have been utilized to improve lymph node trafficking of small molecule adjuvants to the lymph node (134)

BioNTech have developed a lipid-based nanoparticle formulation called Lipoplex. Lipoplex has been used to provide efficient targeting of RNA to dendritic cells. The optimized Lipoplex:RNA formulation uses a charge ratio of 1.3:2, this was found to effectively target RNA to the spleen, form monodisperse and stable particles and was fully resistant to degradation by mouse serum at 37°C (135). In addition to targeting APCs, liposomes can also protect the RNA to be delivered from extracellular ribonucleases and mediates efficient uptake and expression by DCs and macrophages located in various lymphoid compartments. Lipoplex complexed with RNA encoding tumor antigens has also been shown to induce strong effector and memory T cells responses and mediate IFNα dependent rejection of progressive tumors (135). Vaccines using Lipoplex complexed with RNA induce and mobilize both the adaptive and innate immune responses mimicking an antiviral response.

Sahin et al. (136) recently conducted at phase 1 trial (NCT02410733) in melanoma patients who received melanoma FixVac (BNT111) an intravenously administered liposomal RNA (RNA-LPX) vaccine that targets four tumor associated antigens (NY-ESO-1, MAGE-A3, tyrosinase and TPTE) (135). Interim analysis (136) has shown that melanoma FixVac when used alone or in combination with the checkpoint inhibitor PD-1 mediates durable responses in patients with unresectable melanoma and with prior experience with a checkpoint inhibitor. FixVac induced clinical responses and potent CD4+ and CD8+ T cell responses could be detected with the cytotoxic T cell responses in some patients reaching the same level as reported for patients on T cell therapy trials. The completion date for this trial is estimated for December 2021, so far 119 patients (as of August 2020) have been enrolled.

### Self-Assembling Peptides

Self-assembling peptides can also be used as a delivery system to deliver antigens to target cells. Self-assembling peptides can spontaneously form into ordered structures in response to changes in pH, solvent, co-assembling molecules, temperature and ionic strength (137, 138). They can have a diverse range of properties and can be manufactured to form nanomicelles, nanovesicles, nanofibers, nanotubes, nanoribbons and hydrogels (139). Self-assembling peptide deliver systems have a number of advantages over liposomes or nanoparticles including high drug loading, low drug leakage, biodegradability and are highly permeable to target cell membranes. The particle size is important for vaccine delivery and can impact the efficiency of uptake by APCs, with smaller particles (20-200 nm) being more immunogenic but there is no optimal size and this should be optimized for each vaccine candidate (140–142). The smaller particle size is thought to improve uptake into DCs and also the lymphatic system, in addition to size the shape, stability and ability to display multiple antigen can also improve immunogenicity. Self-assembling peptides can be designed to provide vaccines with the desired properties to enable efficient delivery to the target cell.

A delivery system based on modified cell penetrating peptide (CPP) based gene vectors, the Glycosaminoglycan (GAG)-binding enhanced transduction (GET) delivery system has been used to enhance delivery of nucleic acids for lung gene therapy and bone regeneration *in vivo* (143, 144). The GET peptides (143–150) are multi-domain sequences comprising of a heparan sulphate (HS) cell targeting sequence fused to a CPP for improved membrane association and synergistically enhanced intracellular delivery of therapeutic cargoes (145). GET peptides can deliver self-reporting cargo (monomeric red fluorescent protein; mRFP) into difficult to transduce cell types including mesenchymal stem cells (MSCs), human embryonic stem cells (hESCs) and human induced pluripotent stem cells (hiPSCs). Delivery involves a heparin sulphate (HS) cell targeting system fused to a CPP and an endosomal escape peptide and a system which stabilizes particles to prevent aggregation and promote diffusion and cell uptake by PEGylation. The tripeptide complexes the DNA into nanoparticles and can be delivered by simple intramuscular injection. This tripeptide formulation has achieved exceptional results in DNA delivery applications in particular in lung, brain, and has huge potential in vaccine delivery.

## Genetic Vaccines

### DNA Vaccines

DNA vaccines have a number of advantages, they are simple to design, relatively low production costs, good stability (stable at +2-8°C) and solubility, can be rapidly modified, it is a versatile platform that can have many applications including in infectious diseases and oncology. DNA vaccines were first shown to be immunogenic in the 1990s (151–153), they are an attractive cancer vaccine platform (154) and this led to a flood in preclinical and clinical trials. Plasmid DNA vaccines can be designed to act as both an antigen and adjuvant (155), unmethylated DNA containing cytosine-guanine rich regions can act as an adjuvant stimulating an immune response (156). DNA vectors have a negatively charged backbone and have a low molecular weight, therefore naked DNA often suffers from poor cellular uptake resulting in poor antigen production (157–159). With a significant improvement in our knowledge of cancer immunology particular the TME and immune suppression the reasons for the failures of early DNA vaccines are now better understood.

In addition to improving the delivery of plasmid DNA the vector itself can also be modified to specifically target epitopes directly to APCs. In preclinical models we have previously demonstrated that a DNA plasmid encoding T cell epitopes within the complementarity determining regions of a human IgG1 antibody (ImmunoBody^®^) (160), when administered with electroporation (EP) stimulates high avidity T cell responses (161). ImmunoBody^®^ works by the direct uptake of the DNA into APCs, it is then transcribed, translated and processed, with epitopes being presented on the cell surface in combination with MHC. ImmunoBody^®^ can also be taken up by both antigen presenting cells and non-antigen presenting cells and the transcribed antibody protein secreted. The secreted antibody is internalized *via* the high affinity FcγR1 receptor (CD64) on antigen presenting cells, it is then processed, and epitopes cross presented on MHC class I. The combination of direct and cross presentation induces T cells with sufficiently high avidity to eradicate established tumors in preclinical models (160). Immunization with ImmunoBody^®^ DNA vectors induces high frequency and avidity of T cell responses that are superior when compared to those induced by immunization with DNA encoding full-length antigen or when using peptides or peptide loaded onto dendritic cells (161–164). The first ImmunoBody^®^ in the SCIB series, SCIB1, targets four epitopes from the melanoma-associated antigens TRP2 and gp100, our second vaccine, SCIB2, incorporates several epitopes derived from the NY-ESO-1 cancer testis antigen. A first-in-human study performed by Patel et al. used SCIB1 ImmunoBody^®^ that incorporated HLA-A*02:01 restricted epitopes from gp100 and TRP-2 in addition to HLA-DR*04:01 and HLA-DR7/DR53/DQ6 restricted epitopes from gp100. In a cohort of 15 melanoma patients SCIB1 was shown to be safe (165). In this trial 7/15 patients had stable disease, 5/20 fully resected patients experienced disease recurrence and 1 patient had measurable disease, all patients were still alive at the last observation time of 37 months. A phase 2 study in melanoma patients receiving pembrolizumab is now recruiting (ClinicalTrials.gov Identifier: NCT04079166).

### RNA Vaccines

The RNA vaccine platform has the advantage that RNA does not integrate into the host cell genome and thus avoids potential safety concerns, it is also quick to manufacture and can encode multiple epitopes. RNA is single stranded and therefore has a built-in adjuvant function through TLR7 and TLR8 stimulation. However, RNA is very susceptible to cellular degradation, to overcome this in clinical trials it has either been injected directly into inguinal lymph nodes or delivered using a nanoparticle delivery system that protects the RNA. RNA is particularly susceptible to degradation by RNases, to improve transfection and avoid degradation many groups have used delivery systems such as nanoparticles and liposomes (135, 166–170). RNA has an advantage over DNA in that it only needs to be delivered to the cytoplasm for translational into protein unlike DNA that needs to enter the nucleus for transcription.

The first clinical trials using RNA was performed by Weide et al. (171, 172) in patients with metastatic melanoma. In a phase 1 trial in 15 melanoma patients (171) the intradermal administration of naked mRNA was shown to be safe. In a phase 1/2 trial (172) 21 patients received i.d. injections of protamine stabilized mRNA coding for Melan-A, Tyrosinase, gp100, Mage-A1, Mage-A3 and survivin; GM-CSF was used as an adjuvant and half the patients also had keyhole limpet hemocyanin added to the vaccine. The number of clinical responses to the vaccine in this trial was low with only 1 promising clinical response observed in a patient with measurable disease. In a phase 1/2 trial performed by Rittig et al. in 30 patients with stage IV renal cell cancer (173), naked mRNA coding for TAA’s was administered intradermally. This trial demonstrated that vaccination was safe and well tolerated and induced clinical responses in 16 patients; this trial also demonstrated that vaccination induced CD4 and CD8 T cell responses as determined by IFNγ ELISpot and Cr-release assays. The results from these trials demonstrated that vaccination with RNA was feasible and safe. With improvements to trial design, frequency, and route of administration the RNA vaccine platform is progressing through clinical trials in the fields of cancer and infectious diseases. For COVID-19 two RNA vaccines (Tozinameran from Pfizer–BioNTech and mRNA-1273 from Moderna) have now been approved by national health regulators.

In a study performed by Sahin et al. the use of an RNA vaccine encoding neoantigens was explored (174) in melanoma patients. In this study neoantigens were identified by comparative exome analysis in tumors from thirteen patients with stage III and IV melanoma. Mutations were selected for incorporation into the vaccine based firstly on the predicted binding score for HLA class II and secondly based on the predicted binding score for HLA class I. For each patient two synthetic RNAs were synthesized incorporating the identified mutations. The RNA vaccine was produced within 68 days (range 49 to 102 days), following analytical testing they were released within 103 days (range 89 to 160 days). RNA vaccines work in a similar way to the long peptide vaccines, the RNA is translated into protein which is then processed into long peptides by APCs, these peptides are then loaded onto MHC class I or class II molecules and presented on the cell surface to prime and activate T cells. This study demonstrated the clinical feasibility and safety of RNA neo-epitope vaccines. In this study 8/13 patients had no tumors develop during the monitoring period and neoantigen specific T cells could be detected in the peripheral blood of these patients. The use of many neoantigen epitopes in a vaccine reduces the risk of single antigen loss variants (175), however, in this study the outgrowth of B2M deficient tumor cells in one patient demonstrates the complexity of the TME and the selective pressures that drive resistance to therapy.

## Viral Vector Vaccines

Viral vectors have been used in both the gene therapy and vaccine fields. Viral vectors have the advantage of being recognized as foreign by the immune system, inducing potent innate and adaptive immune responses resulting in the induction of strong and durable immune responses. Viral vectors enable the presentation of intracellular antigens incorporated into the vector such as cancer antigens, viral antigens, or a specific gene for gene therapy.

The most commonly used viral vectors are derived from adenoviruses, poxviruses and alpha viruses. The majority of viral vectors are replication defective or attenuated versions, these are preferred from a safety point of view. Viral vectors have a very good safety record with many approved in the infectious disease field such a recently approved Ebola vaccine and COVID-19 vaccines that use adenovirus virus vectors. A disadvantage of the viral vectors is their ability to also induce immune responses that also neutralizes the vector preventing further repeat immunizations. Pre-existing immunity to measles and adenovirus can be problematic limiting the effectiveness and ability to boost responses when adenovirus or measles virus vectors are used. A prime boost vaccine regime is commonly used, and a number of different strategies have been used to overcome the problem with pre-exiting immunity. Strategies using a non-human specific virus such as the replication-defective chimpanzee adenovirus (ChAd68 serotype), or using different vectors derived from different viruses for the prime and boost immunization or using different vaccine platforms for the prime and boost immunizations can all avoid problems associated with pre-existing immunity to the virus vector. A common combination is the use of a DNA prime and a viral vector boost. Another commonly used combination is the Modified Vaccinia virus Ankara (MVA) and Adenovirus (Ad) vectors, both vectors induce potent immune responses that when used in combination in a prime boost regime these responses are further enhanced (176, 177). These strategies have all been used successfully in the infectious disease field, particularly more recently to target SARS-CoV-2, where 40 viral vector vaccines are currently being assessed in preclinical studies, an additional 19 vaccines are currently in clinical trials and another 4 vaccines have already received approval from regulatory authorities (178).

A number of different viral vectors have been used for cancer vaccines (179); with some having progressed into clinical trials. In clinical trials the efficacy of cancer vaccines using viral vectors have not delivered the same results as those generated in the infectious disease field. The immunosuppressive TME and selection of the best cancer antigen to target is problematic and impacts all cancer vaccine platforms. To overcome central tolerance and the immune suppressive TME a cancer vaccine would need multiple boosts in order to induce and sustain a potent immune response, however, this can be problematic due to anti-vector immunity. In preclinical and clinical studies, a prime-boost approach using a recombinant vaccinia vector and a recombinant avipox virus have been successfully used, and multiple boosts using recombinant avipox such as fowlpox is possible. Preclinical and clinical studies have shown that multiple booster vaccinations using a fowlpox does not induce host anti-vector immune responses (180, 181). Both viruses have been shown to be safe, vaccinia was used in the smallpox vaccine that has been delivered to over 1 billion people worldwide. Avipox is an avian virus that is unable to replicate in mammals. Both vectors do not integrate into DNA and successfully infect APCs thus stimulate potent immune responses.

The TRICOM vaccine platform uses the recombinant vaccinia virus (rV-) for the prime and recombinant avipox (fowlpox, rF-) for multiple booster vaccinations. Each vector contains one or more TAAs and transgenes for the costimulatory molecules CD80, ICAM1 and LFA-3. In a phase 2 clinical trial, 125 men with metastatic castration-resistant prostate cancer received a vaccinia virus encoding PSA in combination with GM-CSF followed by six subsequent boosts using a fowlpox virus encoding PSA (PROSTVAC-VF) (182). The results from this phase 2 trial was encouraging with a 10-month improvement in overall survival compared to the empty vector control group (183). Unfortunately, these results were not seen in a large phase 3 study and the study was subsequently stopped (184). It is likely that despite the activation of specific T cells they were either not potent or unable to overcome the immunosuppressive TME (184). Trials are now ongoing to see if combining PROSTVAC-VF/TRICOM with check point inhibitors can improve clinical responses (NCT02933255, NCT04020094, NCT03532217, and NCT03315871).

## Peptide Vaccines

**Figure 3:**
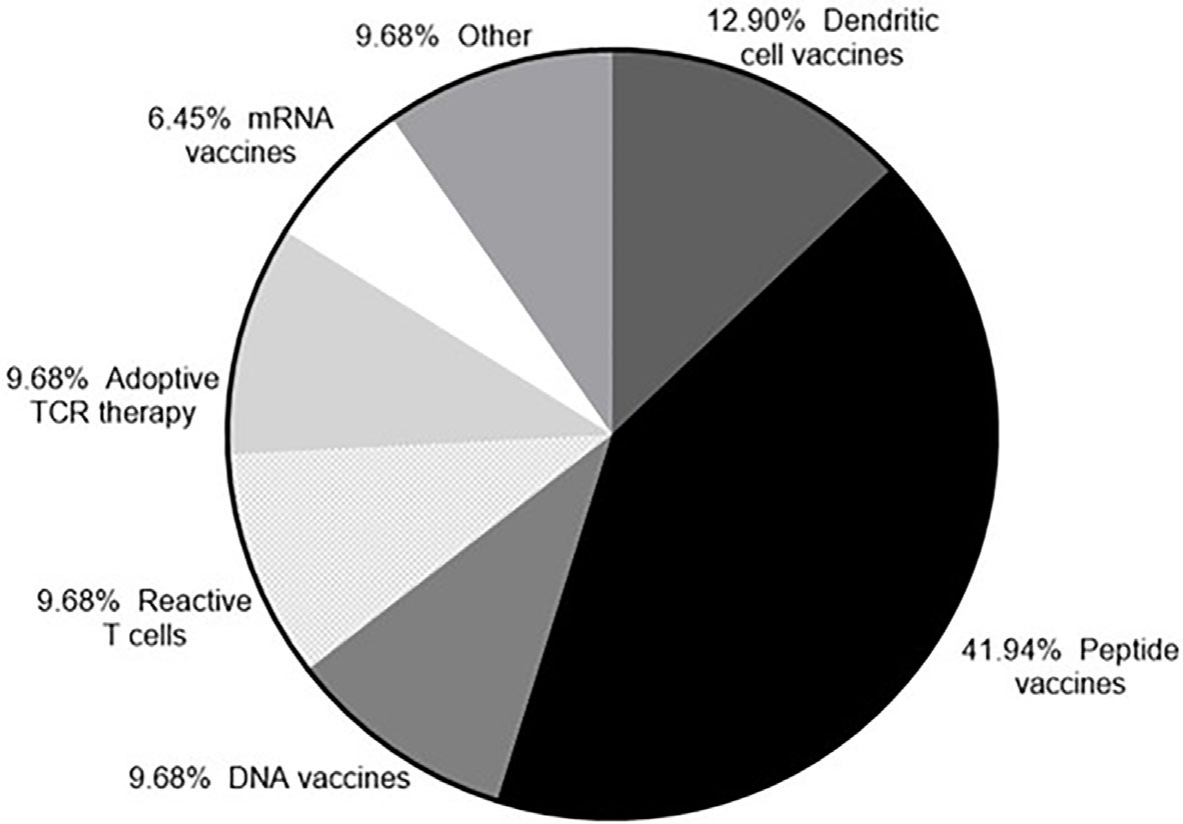
Neoantigens currently in clinical trial. According to clinicaltrials.gov (as of 23^rd^ September 2020) there are currently 33 clinical trials recruiting that target neoantigens.

The number of peptide vaccines being explored has increased due to the discovery of neoantigens. Targeting neoantigens is a personalized therapy and the rapid synthesis of peptides makes the peptides vaccines an attractive platform. Almost half of the clinical trials currently recruiting (as of August 2020) that target neoantigens are using peptide vaccines, with the RNA and DNA vaccine platforms also represented (**Figure 3**). Following administration the peptides included in a vaccine need to be presented on antigen presenting cells (APCs) in order to trigger an adaptive immune response. To efficiently prime an immune response the coadministration of an adjuvant is required to activate the immune system to kill tumor cells expressing the peptide (185–187). Tumor antigens need to be processed and the resulting peptides presented on the cell surface in association with MHC class I or class II molecules. Cancer specific T cells in the TME need to recognize the relevant peptides and kill tumor cells expressing them. The key to the success of a peptide vaccine relies on the correct choice of peptides to include and the best adjuvant to use to generate a local immune response and promote antigen trafficking to local draining lymph nodes. Bioinformatic applications and algorithm prediction program are commonly used to define peptides capable of binding MHC I or MHC class II molecules. Identification of peptides bound to the MHC molecules on the cell surface can be achieved *via* mass spectrometry (MS) analysis. Combining data from MS analysis, epitope predicting algorithms and gene expression data help to predict the best candidate peptides to include in a vaccine.

A vaccine needs to stimulate both CD4+ and CD8+ specific T cells. The majority of peptide vaccines use longer peptides typically 20-30mers, these are likely to contain nested CD8+ T cell epitopes in addition to longer CD4 T cell epitopes and therefore are able to stimulate both CD4+ and CD8+ T cells. In addition, multi-peptide vaccines are often used, incorporating many peptides meaning that many antigens can be targeted, increasing the chances of overcoming any antigen loss on the tumor cells. A number of peptide vaccines targeting neoantigens have been developed by a number of groups, this personalized therapy can target a patient’s individual tumor. Ott et al. (188) used whole-exome sequencing (WES) and RNA-sequencing (RNA-Seq) to identify neoantigens from six stage III/IV melanoma patients. Using NetMHCpan (v2.4) a list of peptides that bind to MHC class I was generated, synthesized peptides were between 15 to 30 amino acids in length and thus capable of stimulating CD4+ and CD8+ T cells. Patients were immunized with 30 peptides given in combination with poly-ICLC. This vaccine-induced polyfunctional CD4+ and CD8+ T cells targeting 58/97 and 15/97 neoantigens respectively across the patients. At 25 months post vaccination 4 patients had no recurrence while 2 patients had progressive disease that was successfully treated with anti-PD-1 therapy.

A number of early peptide-based cancer vaccine trials primarily focused on short peptides from tumor associated antigens, and did not include any delivery system or longer peptide formulations (189). The majority of these trials failed when reaching phase 3 trials, a number of additional reasons for these failures include adjuvant selection, timing of vaccination and peptide formulation (120). Peptide vaccines do generate an immune response but as a monotherapy they can struggle to show efficacy in the clinic. Kimura et al. immunized 39 patients with premalignant colon adenomas with a MUC1 peptide vaccine, they showed the peptides were immunogenic however in 22 patients there was a lack of a response which correlated with a high number of myeloid derived suppressor cells in the TME pre vaccination (190). In another two trials in melanoma and ovarian cancer patients (191, 192), a mixture of peptides were used to immunize patients, vaccination induced an immune response which was associated with some favorable outcomes. The majority of peptide vaccines did not generate an immune response that was robust enough to see any significant clinical benefit (189). These early studies highlighted the need to further optimize peptide vaccines, either by better targeting, better adjuvants or used in combination to overcome the immunosuppressive TME.

### Peptide-Adjuvant Conjugate Vaccines

The administration of free adjuvant with antigens in a cancer vaccine can result in their dissociation following injection and subsequently do not enter the lymphatic system. Adjuvants can be rapidly degraded (193) reducing the amount that reaches the target cells resulting in suboptimal antigen priming and immune response. Free adjuvant in the circulation can also induce autoimmunity (194) or toxicity. The co-delivery of adjuvant with antigens is required to induce a potent immune response while avoiding any autoimmunity or toxicity, as already described a delivery system can be used to deliver the adjuvant and antigen, alternatively the adjuvant and antigen can be linked to improve targeting.

The direct conjugation of a peptide to an adjuvant is gaining increasing attention, particularly from groups targeting neoantigens. Previous reports have described the direct conjugation of peptides to TLR ligands can enhance the immune response by directly targeting the peptide and adjuvant to the same APC (195–199). Peptides can be linked to hydrophobic carriers such as lipids (92), fatty acids (200) and TLR agonists (196, 201, 202) for more efficient delivery to APCs and subsequently lymph nodes. A recent preclinical study performed by Lynn et al. (203) used a peptide platform based on charge modified TLR-7/8a peptide conjugates, these conjugates self-assemble into nanoparticles of uniform size which is independent of the peptide antigen composition. This platform is used to conjugate identified neoantigen peptides, these peptides would possess a variety of properties, such a platform would be able to incorporate peptides with a wide range of characteristics. Neoantigen peptides were predicted (179 peptides) from three murine tumor models, vaccination of mice induced a CD8+ T cell response with approximately 50% of the peptides being recognized, this led to enhanced tumor clearance.

We have directly conjugated citrullinated peptides from vimentin and α-enolase (Vim28-49cit, Vim415-433cit and Eno241-260cit) to the TLR1/2 ligand AMPLIVANT^®^. The direct linkage of a TLR agonist to a peptide can enhances the immunogenicity of the vaccine (196–199). In HLA-DR4 and DP4 transgenic mice vaccination with the conjugated peptides induced a high frequency of specific T cells. The direct conjugation of the Vim28cit, Vim415cit and Eno241cit peptides to the TLR1/2 ligand Amplivant^®^ reduced the peptide-equivalent dose required to induce immune responses by at least 1 log without the loss anti-tumor responses (60). These results demonstrate that the linkage of a TLR ligand to a peptide enhances the immune response and supports the development and application of these peptide/TLR ligand linked conjugates in a clinical setting.

Peptide vaccines have many advantages, they can be chemically synthesized, manufactured at large scale and cost effective (204) when compared to other cancer therapies. In pre-clinical and clinical studies they have been shown to be safe and well tolerated (204, 205). Peptide vaccines should include peptides that target multiple antigens to generate a polyclonal antigen T cell response (206–208). Like DNA and RNA vaccines the use of a delivery system can help improve the targeting and stability of peptides used in a vaccine which reduces any potential off target effects (209–212). The production of conjugated peptide vaccines can be problematic for some peptides, particularly hydrophobic peptides that tend to form aggregates that complicate manufacturing and when injected they form injection site depots leading suboptimal immune responses (213). The conjugation of peptides with adjuvants improves the delivery of both components to APCs which may be needed to induce T cell priming (203, 214). Peptide conjugates used in combination with other therapies have a huge potential. A peptide conjugate vaccine can specifically target APCs and that has the advantage of being dose sparing, in combination with a checkpoint inhibitor to relieve immunosuppression in the TME this would provide the best opportunity for these vaccines to work in the clinic.

## Vaccines in Combination With Other Therapies

Immune checkpoint inhibitors constitute an important breakthrough positively influencing treatment outcomes in cancer patients. Cancer vaccines have the potential to induce potent immune responses but are hampered as tumor cells possess a variety of immune evasions mechanisms that interfere with the function of T cells (215–220). Upon activation, T cells migrate and accumulate in the TME where they can induce tumor cell killing, however, tumors have evolved multiple mechanisms that can dampen or inhibit T cell mediated killing. Tumor cells can alter the antigen processing machinery, secrete immunosuppressive factors that kill the T cells or activate pathways that induce tolerance rendering any tumor therapy ineffective (221). The identification of the key regulators of the immune response has led to the generation of new therapies that have the potential to reverse some of the immune suppression in the TME. Immune checkpoint inhibitors are cell surface receptors that regulate the immune response, they enable self-tolerance while preventing over activation of the immune system resulting in autoimmune disease (222). In the TME the expression of checkpoint receptors suppresses T cell activation and thus provides the tumor with a growth advantage (223). The cytotoxic T lymphocyte protein 4 (CTLA-4) and programmed cell death protein 1 (PD-1) are the best characterised checkpoint receptors in the immunotherapy field, others have been described that are emerging from preclinical studies and entering the clinic.

The checkpoint receptor, PD-1, is expressed on activated T cells and overexpressed on exhausted T cells (90). There are two PD-1 ligands, PD-L1 and PD-L2, PD-L1 is expressed on many cells including immune cells, epithelial cells, endothelial cells and tumor cells (88, 91). PD-L2 is expressed on professional antigen presenting cells including DCs and macrophages (224). The binding of PD-1 on T cells to PD-L1 expressed on a tumor cell or PD-L2 expressed on an APC leads to TCR downregulation, resulting in lower secretion of TNF-α, IFN-γ, and IL-2 (92). The expression of CTLA-4 is induced upon T cell activation and competes with the costimulatory molecule CD28 for its co-stimulatory ligands. CTLA-4 suppresses the early activation of naïve and memory T cells by competing with CD28 (88–90), PD-1 inhibits T cell function at a later activation stage by down regulating TCR expression. Monoclonal antibodies that block CTLA-4 (223), PD-1 (225) or PD-L1 (226) pathways remove the inhibition of T cell function (227) and have made significant clinical impacts. Antibodies that specifically block the CTLA-4 or PD-1/PD-L1 pathway have the potential to remove T cell immune suppression enabling the successful recognition and killing of tumor cells.

Checkpoint blockade has shown promising results in clinical trials and have gained approval for an increasing number of cancers including melanoma, renal-cell carcinoma (RCC), advanced non-small-cell lung cancer (NSCLC), classic Hodgkin’s lymphoma (HL), bladder carcinoma, Merkel cell carcinoma, head and neck cancer, and more recently, solid tumors with mismatch repair-deficiency. PD-1 and CTLA-4 inhibit T cell responses at different stages and by different mechanisms, it is therefore tempting to block both pathways in order to overcome immune suppression. Clinical trials in melanoma patients that have combined PD-1 and CTLA-4 blockade have shown improved clinical responses, however, these have come at a cost with an increase in toxicities being reported (228–230). The combination of anti CTLA-4 and anti PD-1 is now approved as the first line therapy for advanced melanoma patients; however, the toxicities have limited the use of this combination, trials are ongoing to vary the dose and interval of dosing to reduce toxicity.

Many cancer vaccines currently in clinical trials are combined with a checkpoint inhibitor such as CTLA-4, PD-1 or PD-L1 inhibitors, which are offered as standard treatment for an increasing number of cancers. A couple of comparative studies have shown that the combination of a tumor vaccine with a checkpoint inhibitor is more effective than monotherapy (231, 232). Less than 50% of patients respond to checkpoint inhibitors (233–235), there are several other factors that can lead to immune suppression in the TME, such as the action of T-regulatory cells, myeloid-derived suppressor cells (MDSCs), tumor associated macrophages and immunosuppressive DCs (236). For a vaccine to show efficacy in the clinic it is likely a combination with another form of therapy is required to improve tumor specific T cell function in the TME.

**Figure 4:**
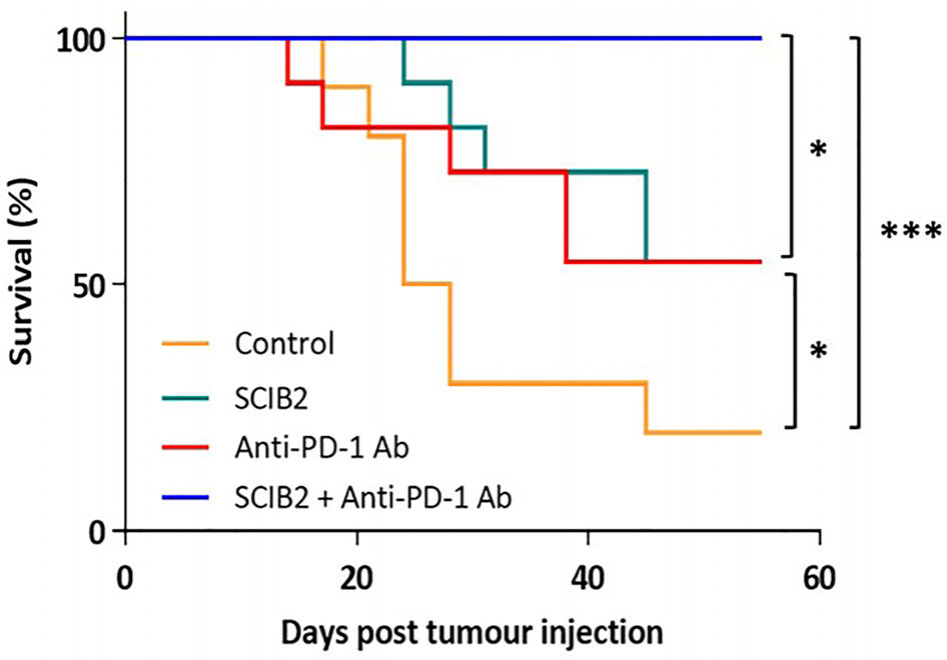
Survival of HHDII mice challenged with 5 x10^4^ tumor cells and immunized with SCIB2 and anti-PD-1 antibody alone or in combination. Control vs SCIB2 (*p = 0.037); Control vs anti-PD-1 antibody (p = 0.111); Control vs SCIB2 and anti-PD-1 antibody (***p = 0.0003); SCIB2 vs SCIB2 and anti-PD-1 (* = 0.0177); anti-PD-1 antibody vs SCIB2 and anti-PD-1 (*P = 0.0177). Lack of survival was defined as tumor size > 528 mm^3^. Each curve represents at least 10 mice per group.

In preclinical murine models using the ImmunoBody® vaccine SCIB2 a synergistic effect was observed when given in combination with anti-PD-1 (**Figure 4**) (237). The synergistic effect was also observed with SCIB1 when given with anti-PD1 (163, 237). These results demonstrate that a cancer vaccine on its own will not achieve the expected results in the clinic without combining with other therapies aimed at modifying the TME by reducing immune suppression and also improving T cell trafficking into the tumor.

The first line treatment for the majority of cancer indications is radiotherapy or chemotherapy. These traditional treatments are not targeted therapies but the damage they cause to tumors results in the release of more antigens from the tumor cells. Damage to tissue surrounding a tumor can also induce the recruitment of T cells into the vicinity, this is particularly valuable when the tumor mutational burden (TMB) is low (238). A number of clinical trials are using either radiotherapy or chemotherapy to enhance the immune response to a vaccine. Radiotherapy can enhance the recruitment of T cells into tumor tissue and increase the intensity of specific anti-tumor immune responses (239). Other studies have shown that some chemotherapeutic drugs can enhance the antitumor activity of tumor vaccines (140, 141) and adoptively transferred T cells (137, 138). A vaccine combined with immune checkpoint inhibitors or traditional treatments can induce stronger anti-tumor responses (188), as such the majority of tumor vaccines in clinical trials are in combination with other therapies. The majority of cancer patients will be offered first line standard of care treatment prior to being offered alternative therapies or participation in a clinical trial.

## Tumor Microenvironment and Tumor Induced Immune Suppression

Tumors evolve and change, they are heterogenous and genetically unstable. Tumors generate many mutations overtime, some are cloned, altered or lost in the tumor genome. Advances in sequencing technology now allows the analysis of a resected tumor or biopsy, the data gathered is a snapshot of a single tumor or part of a tumor at a specific time and does not provide information regarding the overall heterogeneity in the tumor (240, 241). This is a particular problem with the development of a personalized vaccine targeting a neoantigen, the information gained *via* biopsy or resection may represent a mutated tumor subclone or the neoantigen may be not expressed in the whole tumor or metastatic tumors compromising the effectiveness of the vaccine (242). The ideal mutations to target are driver mutations, these are critical for the growth of the tumor and are usually expressed in every tumor cell. However, the number of driver mutations can be low, for example in melanoma only 8% neoantigens are driver mutations (243). The degree of tumor heterogeneity will vary between patients, indications, and tumors. Improving our understanding of tumor heterogeneity will help identify the best epitopes to include in a cancer vaccine and targeting more than one antigen with help overcome tumor heterogeneity. The cancer vaccines targeting neoantigens address this heterogeneity by targeting more than one neoantigen and also addressing potential antigen loss.

Another factor that significantly contributes to the success immunotherapy is the TMB, studies by Rooney et al. demonstrated that the TMB correlated with immune responses (244). Tumors with high TMB, such as melanoma and NSCLC have a higher response rate to immunotherapy compared to tumors with a low TMB, however, this is not the case with all tumors. Pediatric tumors generally have fewer somatic mutations, a study performed by Zamora et al. showed that tumors from children with acute lymphoblastic leukemia had a low TMB but they could still induce a strong anti-tumor response (245). A number of clinical trials are underway for cancer indications that have an unmet need, poor survival and also have a low TMB e.g. glioblastoma and pancreatic cancer. Hopefully the results from these trials will help with our understanding of how to target tumors with a low TMB.

The TME consists of many different cell types including immune and stromal cells, vasculature, extracellular matrix and a variety of cytokines and chemokines. The extracellular matrix is made up of cells from endothelial, mesenchymal and haematopoietic origins. Changes in the TME impact the trafficking of TILs and efficacy of cancer vaccines that have induced specific T cells but are unable to traffic into the tumor.

Tumors have a number of mechanisms that have evolved to suppress anti-tumor immune responses. Apart from checkpoint mediators such as PD-1 and CTLA-4 a number of cell types have been identified that contribute to immune tolerance and evasion in the TME, Myeloid-derived suppressor cells (MDSCs), T regs, tumor associated macrophages (TAMs) and cancer associated fibroblasts all contribute to this immune suppression (246). MDSCs are a type of regulatory cell that are found within the TME (246, 247), they produce nitric oxide, cytokines and reactive oxygen species that can suppress T cells. MDSCs play a critical role in tumor invasion, metastasis and angiogenesis (248, 249). The presence of MDSCs in the TME correlates with poor overall survival and progression free survival (250). In a murine model of rhabdomyosarcoma, the trafficking of MDSCs was inhibited and subsequently an enhanced response to anti-PD-1 therapy was observed (251). In addition to MDSCs the presence of Tregs in the TME in many cancer types is also associated with a poor prognosis (252). Tregs are critical for the maintenance of cell tolerance, they suppress T cell responses by binding IL-2 therefore limiting the amount of free IL-2 available to drive T cell proliferation and activation (253). Tregs express CTLA-4 and can produce immunosuppressive cytokines that further contribute to the immune suppressive TME (252). The TAMs, in particular the M2 macrophages are another cell type that can contribute to the immune suppressive TME (254). The M2 macrophages can promote tumor growth by stimulating tumor cell motility, angiogenesis, and immune evasion (255). Murine studies have demonstrated that the depletion of macrophages reduces tumor growth and also by inhibiting the myeloid growth factor signaling pathway in macrophages overcome resistance to checkpoint inhibitors in a pancreatic cancer murine model (256, 257). The depletion or inhibition of MDSCs, Tregs or TAMs all have the potential to improve anti-tumor responses induced by vaccination or a cellular therapy, however, the impact of depleting these cells in the periphery as well as the tumor increases the potential to induce autoimmunity.

Within the TME the most abundant stromal cells are the cancer associated fibroblasts (CAF), these have been shown to play a role in tumorigenesis, angiogenesis, metastasis, drug resistance, immunosuppression, extracellular matrix (ECM), remodeling and maintenance of cancer stemness (258–264). Different subtypes of CAFs exist each capable of secreting a number of cytokines and chemokines such as TGF-β, IL-6, IL-8, IL-13, CXCL_12_, CXCL_14_, and VEGF that inhibit anti-tumor immune responses. Some CAFs also express PD-L1/PD-L2 or produce metabolites or enzymes such as indoleamine-2,3-dioxygenase (IDO), arginase (Arg), adenosine, and tryptase that recruit Tregs, mast cells and TAMs. CAFs can also contribute to the integrity of tumors, they can synthesize components that make up the ECM such as collagen, fibronectin, matrix metalloproteinases and can contribute to ECM stiffness and thus prevent T cell infiltration. The role of CAFs in cancer progression makes them a promising target for cancer therapy. There have been a few studies looking at anti-CAF based therapies, however in a murine CAR T cell-based study targeting the fibroblast marker FAP, toxicity was observed due to expression of FAP on other tissues (265). Other studies are looking at depleting CAFs, blocking their function or altering their function.

Cytokines and chemokines within the TME can also induce an immune suppressive TME and reduce T cells responses. One of the most studied cytokines is transforming growth factor beta (TGF-β). TGF-β signaling has a massive impact in the TME where it can influence cell growth, differentiation and apoptosis while also inhibiting T cells responses and upregulating Tregs (266). In patients with colon cancer TGF-β tends to suppress cell growth but in advanced stages of the disease the presence of cells expressing members of TGF-β superfamily tend to have a poor prognosis. In murine models the inhibition of TGF-β reverses its immunosuppressive effects and improves the activity of T cells also rendering tumors suspectable to treatment with checkpoint blockade (267, 268). In addition to cytokines in the TME, chemokines such as CXCR2 and CXCR4 that bind to MDSCs and Tregs respectively contribute to tumor immune evasion. In murine models the inhibition of CXCR2 and CXCR4 in combination with anti-PD-1 reversed immune evasion (251, 269). Targeting cytokines and chemokines in TME could be a good cancer immunotherapy strategy that will help change the immune suppressive environment by preventing the recruitment and activation of Tregs, TAMs and MDSCs.

Cancer cells can also lose surface antigens as an immune evasion mechanism following natural or therapy induced selective pressure. Antigen loss has been observed for CD19 in acute lymphocytic leukemia and CD20 in chronic lymphocytic leukemia. Antigen loss is a common reason for resistance to therapy and subsequent relapse. To address the problem with potential antigen loss we have targeted two antigens in our Modi-1 and SCIB1 vaccines, with SCIB1 also inducing high avidity T cells that are capable of responding to a lower number of MHC:peptide complexes on tumor cells. In addition to antigen loss, tumors can also decrease MHC class I expression rendering the immune response powerless. The downregulation of MHC class I has been observed in both human and murine tumors (270–272). The majority of early primary tumors express MHC class I but this profile often changes as the tumor progresses and escape immune surveillance (273). The percentage of cancers that have HLA class I loss, total loss, haplotype loss or allelic loss can range from 65-90% (274, 275). We have addressed MHC class I loss by incorporating both MHC class I and class II peptides in our SCIB1 vaccine, our Modi-1 vaccine only includes MHC class II restricted peptides.

## Conclusions

A large number of cancer vaccines have failed to show clinical efficacy, this can be due to the tumor’s own mechanisms of immune evasion and escape that have evolved including antigen loss, MHC loss, the presence of immune suppressive cells or soluble factors in the TME and lack of a robust anti-tumor immune response (276–279) and also due to the inability of the cancer vaccines to induce sufficiently high avidity T cell responses to efficiently destroy tumors. Early cancer vaccines were primarily focused on stimulating CD8+ T cell responses against tumor associated antigens often using short minimal epitope sequences, T cells recognizing these antigens are highly tolerized and subsequently these vaccines fail in the clinic. The incorporation of CD4+ T cell epitopes into peptides, RNA or DNA vaccine platforms is essential to induce specific CD4+ and CD8+ T cell responses. Targeting non self or mutated tumor antigens will induce high avidity T cells responses when delivered with optimal adjuvant and delivery systems, as such the neoantigens and post translational modified antigens currently show the most promise.

Generating a neoantigen vaccine can be costly both in terms of time and money, the peptide vaccines are personalized and require a significant amount of bioinformatics input to generate the best candidate neoepitopes. The Modi-1 vaccine is not a personalized therapy and is broadly applicable to many patients and cancer types, it is cheaper to manufacture when compared to other platforms and in addition has no time delay constraints that is associated with the production of neoepitope vaccines.

The vaccine platform and the delivery systems used have undergone a huge number of improvements over the last decade. Many new adjuvants have emerged or are currently being investigated in order to improve the immune response at the injection site while also increasing antigen trafficking to the lymph nodes. The majority of cancer vaccines are currently using TLR agonists as adjuvants, these have also been conjugated to peptides or included in nanoparticles to improve targeting. Other adjuvants such as STING, CD40 agonist and GM-CSF are currently being investigated in clinical trials. The correct selection of an adjuvant is key to the ability of the vaccine to induce a robust immune response. We have previously screened a number of different potential adjuvants to use in our Modi-1 vaccine, this included CpG (TLR9), MPLA (TLR4), CpG/MPLA (TLR9/TLR4), GM-CSF, imiquimod (TLR7), Poly I:C (TLR3) or TLR1/2 (AMPLIVANT^®^). Preclinical studies have shown that when AMPLIVANT^®^ is given in combination with the Modi-1 peptides it induced the strongest anti-tumor response (60). This highlights the importance of determining the best delivery and targeting approach for a vaccine that generates the strongest immune response while reducing any possible toxicity.

With our ImmunoBody^®^ platform we have modified a DNA vector by engineering T cell epitopes into the IgG1 CDR regions (163), the Fc region of the antibody targets the high affinity Fc receptor CD64 that is expressed on activated APCs. The SCIB DNA vaccines allow both direct- and cross-presentation of epitopes by targeting dendritic cells, and are able to generate high avidity CD8+ T cells that efficiently eradicate tumors.. Vaccination with SCIB1 induces high frequency and high avidity specific T cells (165).

Improvement with vaccine delivery systems has led to the generation of nanoparticles, self-assembling peptides, and needle free delivery systems. Electroporation was used to administer SCIB1 in our phase 1 clinical trial, and has been used to deliver other cancer and infectious disease vaccines. However, the pain on administration using electroporation and the requirement for specialized vaccine delivery instrument have limited their use, these newer delivery systems provide better alternatives. Liposomes are increasingly being used as delivery system, they are versatile, incorporating small drug candidates or antigens in the form of RNA or peptides, and have a good safety profile. Liposomes do require optimization in order to determine the optimal charge/size of the particle to incorporate their cargo and deliver it across the cell membrane. The targeting of cancer antigens to improve the local immune response and trafficking to lymph nodes can be achieved through either the linking of peptide to adjuvant, incorporating the antigen into nanoparticles or by modifying genetic vectors eg ImmunoBody^®^ platform.

There are a number of challenges to address in the development of a successful cancer vaccine, we have tried to address a number of these challenges with our SCIB1, SCIB2 and Modi-1 vaccines. The SCIB1 vaccine is currently in phase 2 trials in combination with anti-PD-1 therapy, and the Modi-1 vaccine will be entering clinical trials in 2021. The success of any cancer vaccine does not rely only on the ability of the vaccine to induce a robust immune response but also on the modification of the immune suppressive TME to enable the successful trafficking of T cells and the ability of these T cells to recognize and kill tumor cells. The tumor size, TMB and previous treatments will all influence the success of a cancer vaccine and this will vary among patients and cancer types. With huge improvements in the cancer vaccine field and a better knowledge of the TME with time cancer vaccines will start to show good clinical efficacy.

## Author Contributions

SP wrote the review article. VB and PS contributed and analyzed the data. LD proof read the review and conceived the ideas and work around the Modi-1 and Immunobody vaccine platforms. All authors contributed to the article and approved the submitted version.

## Funding

This work was funded by Scancell Ltd.

## Conflict of Interest

LD is CSO and shareholder in Scancell Ltd. SP, VB, and PS are employees of Scancell Ltd.

The authors declare that this study was funded by Scancell Ltd. The funder was involved in the study design, collection, analysis, interpretation of data, the writing of this article and the decision to submit it for publication.
